# Role of VEGF Receptors in Normal and Psoriatic Human Keratinocytes: Evidence from Irradiation with Different UV Sources

**DOI:** 10.1371/journal.pone.0055463

**Published:** 2013-01-31

**Authors:** Jian-Wei Zhu, Xian-Jie Wu, Zhong-Fa Lu, Dan Luo, Sui-Qing Cai, Min Zheng

**Affiliations:** 1 Department of Dermatology, The Second Affiliated Hospital, Zhejiang University School of Medicine, Hangzhou, Zhejiang, China; 2 Department of Dermatology, The First Affiliated Hospital of Nanjing Medical University, Nanjing, Jiangsu, China; University of Tennessee, United States of America

## Abstract

Vascular endothelial growth factor (VEGF) promotes angiogenesis and plays important roles both in physiological and pathological conditions. VEGF receptors (VEGFRs) are high-affinity receptors for VEGF and are originally considered specific to endothelial cells. We previously reported that VEGFRs were also constitutively expressed in normal human keratinocytes and overexpressed in psoriatic epidermis. In addition, UVB can activate VEGFRs in normal keratinocytes, and the activated VEGFR-2 signaling is involved in the pro-survival mechanism. Here, we show that VEGFRs were also upregulated and activated by UVA in normal human keratinocytes via PKC, and interestingly, both the activated VEGFR-1 and VEGFR-2 protected against UVA-induced cell death. As VEGFRs were over-expressed in psoriatic epidermis, we further investigated whether narrowband UVB (NB-UVB) phototherapy or topical halomethasone monohydrate 0.05% cream could affect their expression. Surprisingly, the over-expressed VEGFRs in psoriatic epidermis were significantly attenuated by both treatments. During NB-UVB therapy, VEGFRs declined first in the basal, and then gradually in the upper psoriatic epidermis. VEGFRs were activated in psoriatic epidermis, their activation was enhanced by NB-UVB, but turned undetectable after whole therapy. This process was quite different from that by halomethasone, in which VEGFRs and phospho-VEGFRs decreased in a gradual, homogeneous manner. Our findings further suggest that UV-induced activation of VEGFRs serves as a pro-survival signal for keratinocytes. In addition, VEGFRs may be involved in the pathological process of psoriasis, and UV phototherapy is effective for psoriasis by directly modulating the expression of VEGFRs.

## Introduction

It is known that VEGF is expressed by epidermal keratinocytes and is upregulated in wound healing, psoriasis, and other states of increased skin angiogenesis as well as by UV irradiation [1]–[9]. Keratinocyte-derived VEGF was thought to act in a paracrine manner mainly affecting dermal blood vessels and tissues [1], [10]. The targeted over-expression of VEGF in the epidermis of transgenic mice results in enhanced skin vascularization [11], and development of chronic inflammatory skin lesions after an acute induction of skin inflammation [12]. UV upregulates VEGF production in keratinocyte-derived cell lines both directly through transcription factor activation and indirectly through cytokine release [6], [9]. One of the major acute effects of UV exposure is the induction of erythema, cutaneous inflammation, vascular leakage, and edema formation, all of which are characteristics of sunburn. Moreover, the angiogenic response induced by UVB in VEGF transgenic mice contributed to cutaneous photodamage but not part of a physiologic repair mechanism [13].

Although keratinocytes can express several isoforms simultaneously, the most abundant form is VEGF165, which binds several identified receptors, including the transmembrane tyrosine kinase receptors VEGF receptor-1 (VEGFR-1), VEGFR-2, and the cell-surface non-tyrosine kinase receptor neuropilin-1(NRP-1) [14]–[17]. Though VEGFR-1 is also a high-affinity receptor for VEGF, it demonstrates much weaker VEGF-dependent tyrosine phosphorylation than VEGFR-2 [18], so VEGFR-2 is believed to mediate most of its functional effects [19], [20]. NRP-1 has been shown to be an isoform-specific receptor for VEGF165 [21], it acts as a co-receptor by forming complexes with VEGFR-2 and enhancing VEGF binding [22], [23], while it alone is insufficient to mediate VEGF effects [14]. VEGF affects the behavior of epidermal melanocytes, who have been defined to constitutively express VEGFR-1, VEGFR-2 and NRP-1, and their expression of VEGFR-2 was upregulated by UVB irradiation and downregulated by VEGF and TNF-α [24]. We have previously showed that keratinocytes from normal human epidermis also expressed all five known VEGF receptors and co-receptors [25]. Significant effects of the VEGF/VEGFR-2 autocrine signaling pathway have been found in keratinocytes [25], , even in epidermal appendages that are lined with epithelial cells [27]. In addition, we recently showed that moderate doses of UVB enhanced the expression and activation of VEGFRs in keratinocytes, and activation of VEGFR-2 by UVB was involved in promoting cell survival [28].

However, solar ultraviolet reaching the earth is a combination of UVB and UVA wavelengths. Although they share many common features in regards to the effects on human skin, recent evidence suggests that these effects are achieved through differential activation of signaling pathways [29]. Since UVA is less energetic than UVB, UVB has long been thought to be the most damaging factor in the sunlight. But with modern tools such as in vitro models, it has been proven that UVA plays a major role. Even if UVA is less energetic than UVB, it is more abundant and penetrates deeper into the skin, reaching as far as the dermis. Acute as well as chronic sun exposure induces short- and long-term clinical damages. The long-term effects are photoaging and photocarcinogenesis, which is induced by chronic UVA exposure [30]. UVA-damaged cells normally undergo apoptosis, but failed apoptosis might lead to cancer development. Indeed, cell survival can be promoted through survival signals, thus allowing potential malignant transformation, but the molecular mechanisms of cell survival after UVA irradiation are not fully understood.

Herein, in order to further investigate the functional significance of VEGFRs, we examined the expression, activation and potential roles of VEGFRs in normal human keratinocytes in response to UVA irradiation. In addition, as VEGFRs were found to be over-expressed in psoriatic epidermis and might be involved in the pathogenesis of psoriasis [31], and that narrowband UVB (NB-UVB) phototherapy or topical halomethasone monohydrate 0.05% cream has been clinically demonstrated to be effective for psoriasis, we also examined whether the expression and activation of VEGFRs in the psoriatic epidermis could be affected by these two treatments.

## Materials and Methods

### Isolation and culture of normal human keratinocytes

Adolescent foreskin was obtained from urinary surgery and handled aseptically. After removal of subcutaneous elements, the skin was cut into 0.5 cm^2^ pieces and put into 0.5% dispase (Gibco, Invitrogen, Grand Island, NY, USA) for overnight incubation at 4°C. The epidermis was peeled off from the dermis and incubated in 0.25% trypsin for 10 min at 37°C. Trypsin activity was neutralized by fetal bovine serum (FBS). Cell suspension was filtered through nylon gauze (200 µm mesh) and cells were washed twice with 0.1 M phosphate-buffered saline (PBS) prior to resuspension in keratinocyte serum-free medium (KC-SFM) (Gibco, Invitrogen, Grand Island, NY, USA), supplemented with 5×10^−3^ µg/ml epidermal growth factor, 0.05 mg/ml bovine pituitary extracts, penicillin(100 units/ml) and streptomycin(100 mg/ml). Cells were seeded onto 100 mm dishes (Corning, Corning NY, USA), cultured at 37°C in a humid atmosphere containing 5% CO_2_. Passages 3 to 6 were used in all experiments.

### UVA irradiation regime for cultured normal keratinocytes

Confluent and quiescent keratinocytes were washed twice with PBS, irradiated by UVA under a thin film of PBS, and replenished with their own medium after irradiation. The UVA source was a parallel bank of nine PL-S9W/10R fluorescent tubes (Philips, Eindhoven, Holland) delivering a continuous energy range between 320–400 nm [32]. Fluence rate at the site of cell irradiation was 4.15 milliwatts/cm^2^ as measured by a Centra radiometer (Osram, Munich, Germany). The UVA doses ranged from 5 to 25 J/cm^2^ (exposure time: 20–100 min), and was sublethal for cells below 10 J/cm^2^, which is a realistic representation of the irradiation reaching basal keratinocytes in vivo [33]. Aliquots of cells were harvested at different time points or with different doses after irradiation for RNA isolation and protein extraction.

### Human participants

First, 5 healthy volunteers including 3 males and 2 females, aged from 19 to 27 years old (mean 22.8), were enrolled for UVA irradiation. Using solar simulated equipment switched to UVA source (SUV1000, Sigma high-tech, Shanghai, China), minimal erythema dose (MED) was determined individually in the buttocks where they had received no sun exposure at least 3 months prior to UVA treatment. Then, participants were instructed to lie down on their stomach, and a parallel bank of nine UVA source (TL100W/10R) fluorescent tubes was applied to irradiate the buttocks of participants, which was covered with a plastic baffle leaving 2 round holes (1 cm in diameter). Fluence rate at the site of irradiation was 41.8 milliwatts/cm^2^ as measured by a Centra radiometer (Osram, Munich, Germany). They were exposed to single dose of 1 MED (58.35±16.76 J/cm^2^) or 3 MEDs in two sites of the buttocks respectively. The time of a single exposure ranged from 24.6±5.7 min for 1 MED to 73.9±17.7 min for 3 MEDs. Both the two doses could induce an acute skin injury, the former is moderate, and the latter is serious. Skin biopsies (3 sites from each volunteer: sham-irradiated, 1 MED, and 3 MEDs) were taken 24 h after irradiation.

Second, 13 patients with psoriasis vulgaris but without any other disease, including 9 males and 4 females, aged from 17 to 63 years old (mean 41.2), were enrolled for NB-UVB phototherapy. The patients had not received any systemic drug therapy or topically used medications 6 months before and throughout the whole NB-UVB phototherapy. The UVB source was a parallel bank of 32 TL100W/01 fluorescent tubes (Philips, Eindhoven, Holland) fixed in an equipment (SS-05B-32, Sigma high-tech, Shanghai, China), delivering a continuous energy range between 311 and 313 nm. Phototherapy was performed following the irradiation protocol, starting from the dose of half a MED and three times a week respectively, with a dose increase (0.1 kJ/m^2^) for each irradiation. The therapy duration ranged from 1.5 to 3 months and peak UVB dose ranged from 1.5 to 2.2 kJ/m^2^ according to patients' response. The times of treatment in relation to during (psoriatic lesions got improved, with attenuated clinical signs but still not resolved) and after (7 days after the whole therapy with skin lesions completely resolved) therapy were 11.2±2.2 (or 25.5±6.1 days) and 26.3±3.9 (or 63.6±12.0 days), respectively. The overall skin condition were evaluated by 3 independent physicians. Typical lesions that could represent the overall skin condition before, during and after therapy were taken for biopsy from the trunk.

Third, 3 patients with psoriasis vulgaris but without any other disease, including 1 male and 2 females, aged from 28 to 36 years old (mean 31.7), were enrolled for topical use of halomethasone monohydrate 0.05% cream. The enrollment criteria were the same to that for phototherapy. Halomethasone cream was applied to the lesions once a night before sleep. The whole therapy ranged from 24 days to 1.5 months, and skin biopsies were taken during (12.7±2.9 days) and after (36.0±8.0 days) therapy. The overall skin condition were evaluated by 3 independent physicians. Typical lesions that could represent the overall skin condition before, during and after therapy were taken for biopsy from the trunk. All specimens were embedded in optimum cutting temperature (OCT) compound (Tissue-Tek; Miles, Naperville, IL, USA) and snap-frozen in liquid nitrogen until processed.

All participants were recruited to this study from Department of Dermatology, Second Affiliated Hospital, Zhejiang University School of Medicine. All volunteers gave informed written consent. This study was conducted in agreement with the Declaration of Helsinki and was specifically approved by the institutional review committee of Zhejiang University.

### Indirect immunofluorescence

The embedded skin specimens were cut into 6 µm sections using a Leica CM 1850 cryostat (Meyer Instruments, Houston, TX, USA) and placed on the slides. After being fixed with iced acetone for 10 min, the sections were permeabilized with PBS containing 0.1% TritonX-100 for 15 min and blocked in PBS containing 5% bovine serum albumin (BSA) for 1 h at room temperature. Then, the sections were incubated overnight at 4°C with 20 µg/ml primary antibodies against VEGF165, VEGFR-1, VEGFR-2, NRP-1 (R&D Systems, Minneapolis, MN, USA), and P-VEGFR-2 (Tyr1175) (Cell Signaling Technology, Beverly, MA, USA) diluted with PBS containing 1% BSA. After 3 washes with PBS, the sections were incubated with Rhodamine or FITC-conjugated secondary antibodies (Dako-Cytomation, Denmark A/S, Glostrup, Denmark) that was diluted 1∶200 with PBS containing 1% BSA for 2 h in the dark. After 3 washes, the sections were counterstained with 4,6-diamino-2-phenyl indole (DAPI) (Sigma–Aldrich, USA) for nuclei and pictured under fluorescence microscopy (Olympus, BX51, Tokyo, Japan). Negative controls that were stained with non-immune mouse IgG were included in all experiments. All images were analyzed by ImageJ 1.44p software.

### Reverse transcription and polymerase chain reaction (RT-PCR)

Keratinocytes were grown to 70% confluency and treated by UVA. Total RNA was isolated by using Trizol reagent (Gibco, Invitrogen, Grand Island, NY, USA). cDNA was synthesized from 2 µg of total RNA in a reaction volume of 20 µl. Primers were synthesized (Sangon, Shanghai, China) with sequences : for *VEGF165*, 5′-GGCCTGGAGTGTGTGCCCAC-3′ and 5′-CCGCTCTGAGCAAGGCCCAC-3′; for *VEGFR-1*, 5′-ATGGCTCCCGAATCTATCTTTGAC-3′ and 5′-GCCCCGACTCCTTACTTTTACTGG-3′; for *VEGFR-2*, 5′-CTGGCGGCACGAAATATCCTCTTA-3′ and 5′-GGCCGGCTCTTTCGCTTACTGTTC-3′; for *NRP-1*, 5′-AGCCCCTCCTCCTGTTGTGTCTTC-3′ and 5′-GCTATCGCGCTGTCGGTGTAAAAA-3′; for *GAPDH*, 5′-TGAAGGTCGGAGTCAACGG-3′, and 5′-TGGAAGATGGTGATGGGAT-3′. Then PCR was performed as described [28], with 35 cycles. PCR products were fractionated by gel electrophoresis and quantified by Quantity One software (Bio-Rad).

### Western blot analysis

Cells were treated by UVA when grown to 70% confluency. Total cellular protein was extracted and its concentration was measured as described [28]. Cell lysate was boiled in 5×loading buffer (Beyotime, Jiangsu, China) for 10 min and 40 µg of protein was loaded for each lane. Total protein was separated by 10% SDS–PAGE and transferred to PVDF membrane (Piscataway, NJ, USA). Blots were blocked for 1 h in TBST containing 5% BSA. After being rinsed three times for 10 min with TBST, the membrane was incubated with the primary antibodies(1∶500–1∶2000) to P-VEGFR-2 (Tyr1175) (Cell Signaling Technology, Beverly, MA, USA), VEGF165, VEGFR-1, VEGFR-2, NRP-1, P-VEGFR-1 (Y1213), Bcl-2, cleaved Caspase-3, Akt, P-Akt (Thr308), ERK1/2, P-ERK1/2 (Thr202/Tyr204), GAPDH (R&D Systems, Minneapolis, MN, USA), and overnight at 4°C in TBST containing 1% BSA. Blots were then washed 3 times (each 10 min) in TBST and were incubated for 2 h with horseradish peroxidase-conjugated secondary IgG antibody (Jackson, West Grove, PA, USA). The membrane was washed 3 times (each 10 min) with TBST. The immunoreactive bands were detected by using enhanced chemiluminescent (ECL) plus reagent kit (Piscataway, NJ, USA). The results of densitometric scanning of immunoreactivity on each lane were normalized by blotting for GAPDH.

### Apoptosis Assay

Cell apoptosis was evaluated using the Annexin V-EGFP/PI Apoptosis Detection Kit (KeyGen, Nanjing, China), which is based on the translocation of phosphatidylserine from the inner leaflet of the plasma membrane to the cell surface in early apoptotic cells. Briefly, cells were seeded in 6-well culture plates and cultured until 70% confluency. Then, cells were exposed to various doses of UVA, and neutralizing antibodies against VEGFR-1 and VEGFR-2 were applied into the culture medium. Cells were harvested 24 h thereafter and resuspended in binding buffer according to the protocol. Annexin V-EGFP and propidium iodide (PI) were then added to the buffer and incubated for 15 min in the dark, followed by flow cytometry using a fluorescence-activated cell sorter (FACS, Becton Dickinson, USA). The percentage of apoptosis was computed using CellQuest software (Becton Dickinson).

### Survival assay

Cell survival was determined by MTT assay, which determines mitochondrial activity in living cells. Briefly, cells were plated in collagen-coated 96-well plates (5×10^4^ cells/ml, 100 µl/well) and cultured overnight. After treatment with or without 10 J/cm^2^ UVA, cells were cultured by adding neutralizing antibodies against VEGFR-1 or VEGFR-2 for another 24 h. Then, 20 µl of 3-[4,5-dimethylthiazol-2-yl]2,5-diphenyltetrazolium bromide (MTT; Sigma-Aldrich, USA) at a final concentration of 5 mg/ml in PBS was incubated with the analyzed cells for 4 h at 37°C. Dark blue formazan crystals that formed in the living cells were dissolved by adding 100 µl of DMSO. The optical absorbency was measured at 490 nm with a spectrophotometric reader Elx800 (Bio-Tek, Winooski, VT, USA). Results represent the means of three experiments, and cell survival is expressed by the OD value.

### Statistics

Results are expressed as means ± SD. All experiments were performed at least in triplicate unless otherwise indicated. Statistical analysis was performed using one-way analysis of variance (ANOVA), Student's t-test, with a *P* value less than 0.05 considered to be statistically significant.

## Results

### The expression of VEGFR-1, VEGFR-2 and NRP-1 is increased and the phosphorylation of VEGFR-1 and VEGFR-2 is promoted by UVA irradiation in normal human keratinocytes and epidermis

**Figure 1:**
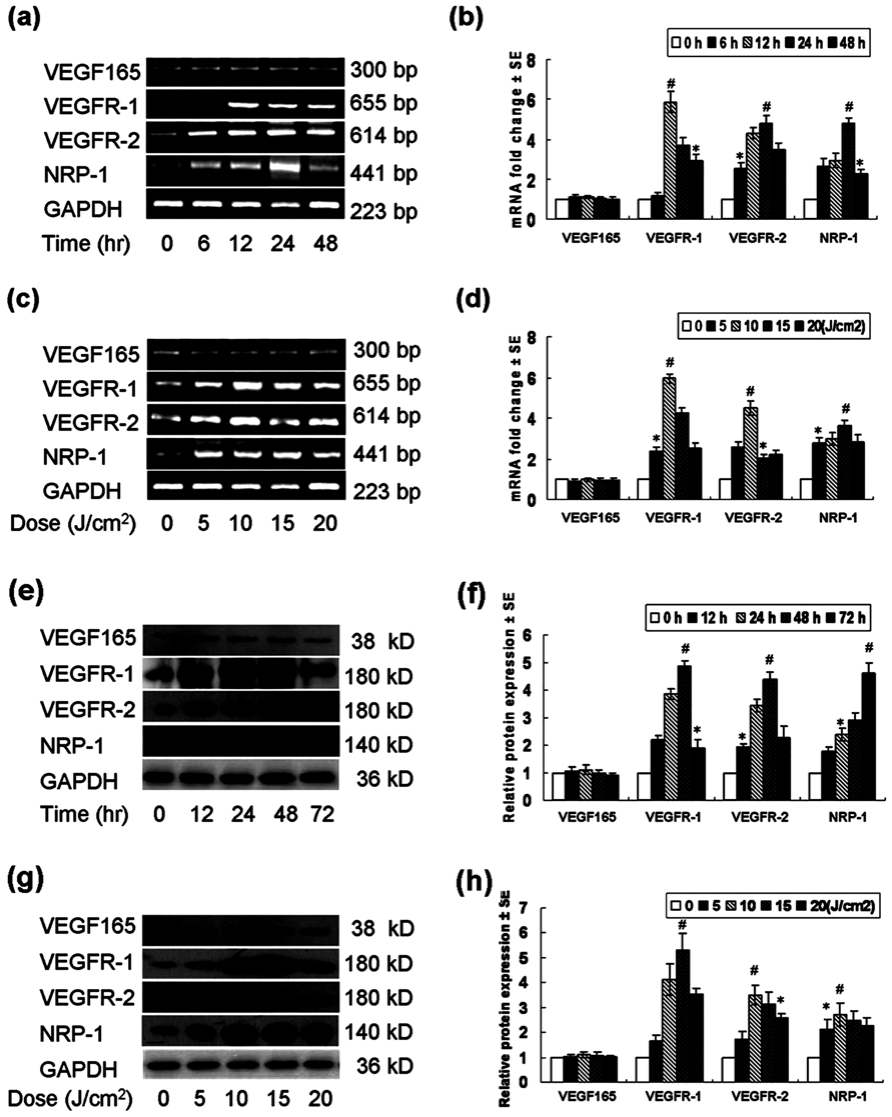
UVA upregulated mRNA and protein expression of VEGFRs in normal human keratinocytes. (a) The time-dependent mRNA expression of VEGF165 and VEGFRs in keratinocytes treated by 10 J/cm^2^ UVA. (b) The relative fold change analysis of (a). (c) The UVA dose-dependent mRNA expression of VEGF165 and VEGFRs in keratinocytes harvested at 24 h after irradiation. (d) The relative fold change analysis of (c). GAPDH was served as an internal control for mRNA normalization. (e) The time-dependent protein expression of VEGF165 and VEGFRs in keratinocytes treated by 10 J/cm^2^ UVA. (f) The densitometric analysis of (e). (g) The UVA dose-dependent protein expression of VEGF165 and VEGFRs in keratinocytes harvested at 24 h after irradiation. (h) The densitometric analysis of (g). The relative protein expression was normalized to the endogenous control GAPDH, * *P*<0.05; # *P*<0.01.

First, mRNA expression of VEGFR-1, VEGFR-2 and NRP-1 was upregulated by UVA irradiation in normal keratinocytes. In the time-dependent study, a significant induction of VEGFR-1, VEGFR-2 and NRP-1 mRNA was detected as early as 6 h and in the highest level 24 h after exposure (Figure 1a & b). In the dose-dependent study, upregulation of VEGFR-1, VEGFR-2 and NRP-1 mRNA was also induced under the smallest dose (5 J/cm^2^), and peaked under 10 J/cm^2^ (VEGFR-1, VEGFR-2) or persisted to be with high expression (NRP-1). None dropped to basal level even under higher doses (Figure 1c & d).

Second, VEGFR-1, VEGFR-2 and NRP-1 proteins were also upregulated by UVA. In the time-dependent experiment, a significant induction of these proteins was detected as early as 12 h and peaked at 48 h (VEGFR-2) or 72 h (VEGFR-1, NRP-1) after irradiation (Figure 1e & f). In the dose-dependent study, upregulation of VEGFR-1, VEGFR-2 and NRP-1 was induced under the lowest dose (5 J/cm^2^), and peaked under 10 or 15 J/cm^2^, and again, none of these receptors dropped to basal level even under high doses (Figure 1g & h).

**Figure 2:**
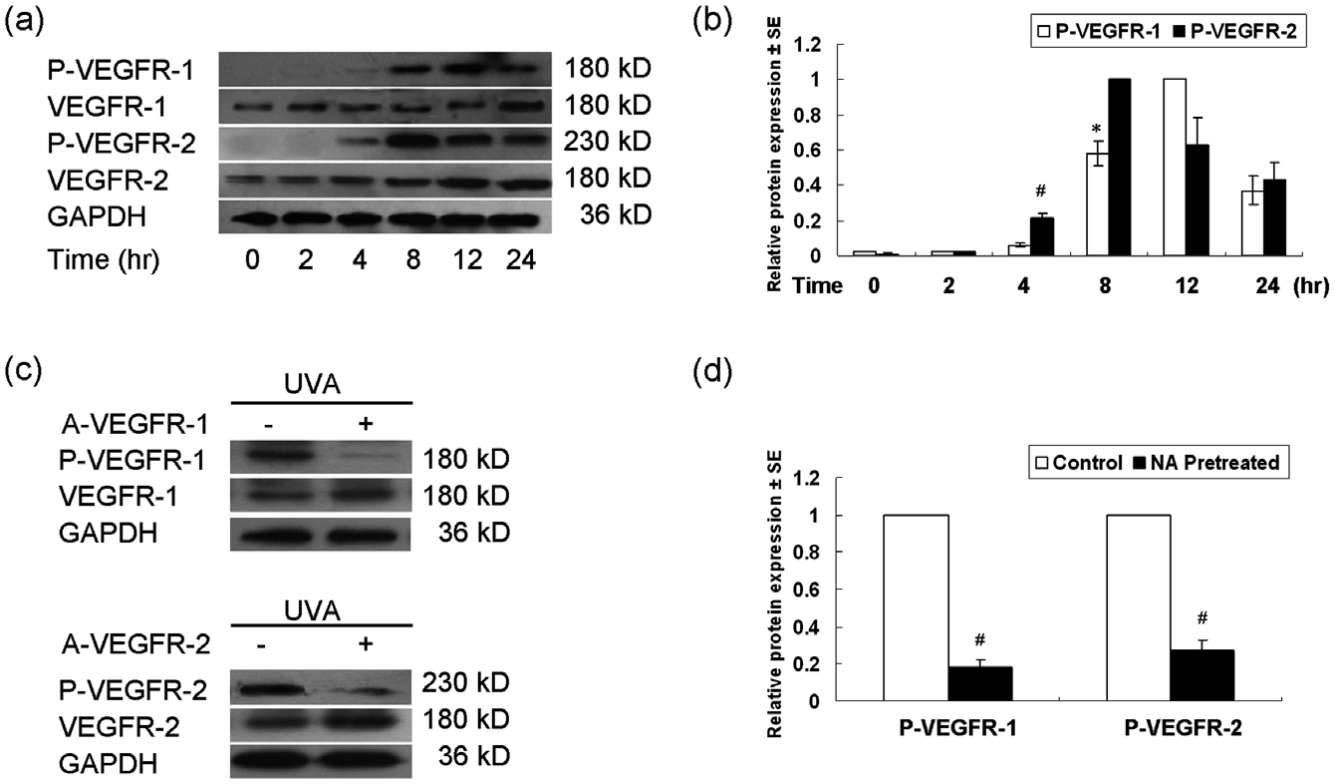
UVA promoted tyrosine phosphorylation of VEGFR-1 and VEGFR-2 in normal human keratinocytes. (a) The time-dependent phosphorylation of VEGFR-1 and VEGFR-2 in keratinocytes induced by 10 J/cm^2^ UVA. Cells were harvested 0, 2, 4, 8, 12, 24 h after irradiation. (b) The densitometric analysis of (a). (c) Top panel: The phosphorylation of VEGFR-1 in keratinocytes 12 h after treatmet of 10 J/cm^2^ UVA with or without pre-incubation of VEGFR-1 neutralizing antibody (A-VEGFR-1, 5 µg/ml) for 1 h. Bottom panel: The phosphorylation of VEGFR-2 in keratinocytes 12 h after treatmet of 10 J/cm^2^ UVA with or without pre-incubation of VEGFR-2 neutralizing antibody (A-VEGFR-2, 5 µg/ml) for 1 h. (d) The densitometric analysis of P-VEGFR-1 and P-VEGFR-2 in (c). The relative protein expression was normalized to the endogenous control GAPDH. NA, neutralizing antibody; P-VEGFR-1, phospho-VEGFR-1 (Y1213); P-VEGFR-2, phospho-VEGFR-2 (Tyr1175); UVA: UVA; * *P*<0.05; # *P*<0.01.

Next, we examined whether UVA could promote the activation of VEGFR-1 and VEGFR-2. As shown in Figure 2a, 10 J/cm^2^ UVA induced the tyrosine phosphorylation of VEGFR-1 8 h (peaked at 12 h) and VEGFR-2 4 h (peaked at 8 h) after irradiation. Incubation with a neutralizing antibody for VEGFR-1 or VEGFR-2 almost completely abrogated their phosphorylation (Figure 2c).

**Figure 3:**
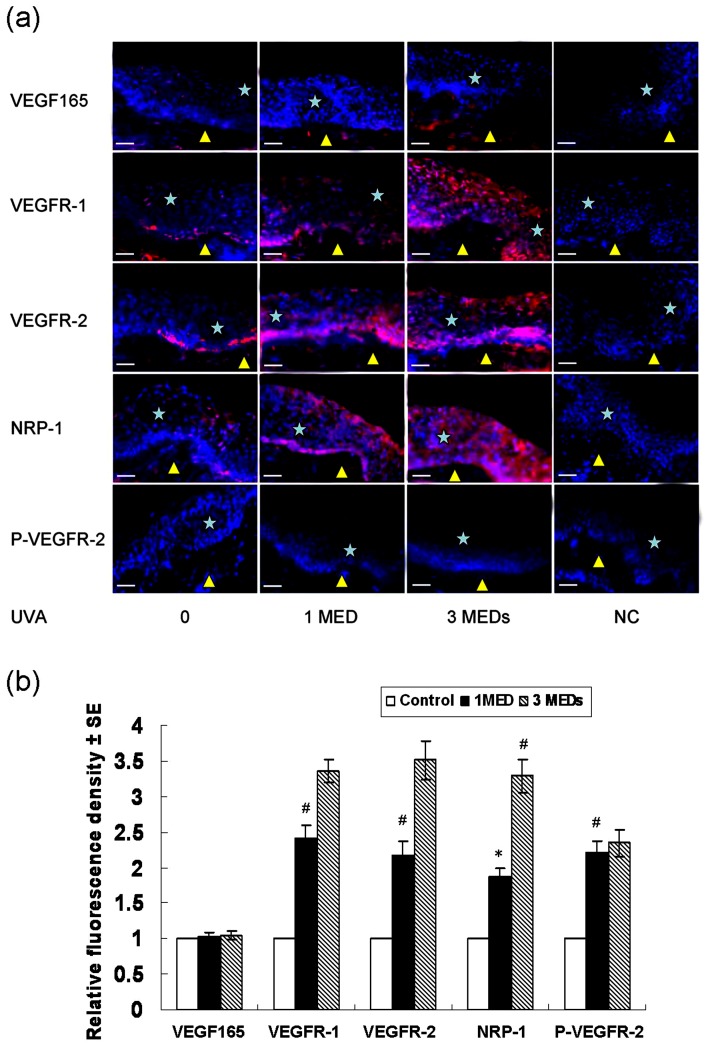
Immunofluorescence detection of VEGF, VEGFRs and P-VEGFR-2 regulated by UVA in normal human epidermis. (a) Expression and localization of VEGF165, VEGFR-1, VEGFR-2, NRP-1 and P-VEGFR-2 by immunofluorescence regulated by UVA in normal human epidermis. (b) The fluorescence density analysis of (a). Skin samples from 5 independent individuals were used for quantification. Biopsies were taken 24 h after treatment of 0, one MED, and three MEDs of UVA respectively. The presence of VEGF165 and VEGFRs was indicated by red fluorescence. The presence of P-VEGFR-2 was indicated by green fluorescence. The cellular nuclei were counterstained with DAPI (blue nuclear signal). P-VEGFR-2, phospho-VEGFR-2 (Tyr1175); UVA: UVA; MED, minimal erythema dose; NC, negative controls, which were incubated with non-immune mouse IgG. Bars: 50 µm; Asterisk, epidermis; Yellow triangle, dermis; * *P*<0.05; # *P*<0.01.

We then asked whether the expression of VEGFR-1, VEGFR-2 and NRP-1 and the activation of VEGFR-1 and VEGFR-2 could be enhanced by UVA in normal human skin. As shown in Figure 3, all of these receptors were expressed in a low level in normal epidermis. VEGFR-1 and VEGFR-2 were distributed mainly in the basal layers, while NRP-1 was homogeneously distributed in all layers of keratinocytes, which was similar to our previous reports [25], [28]. Generally, the expression of VEGFR-1, VEGFR-2 and NRP-1 in the epidermis was significantly enhanced by UVA. NRP-1 was homogeneously increased in all layers, in contrast, the expression of VEGFR-1 and VEGFR-2 was strongly induced in the upper layers, where there was little expression before irradiation. Phosphorylation of VEGFR-2 was also triggered by UVA, and P-VEGFR-2 mainly distributed in the upper layers of the epidermis. Both moderate (1 MED) and high (3 MEDs) doses of UVA enhanced the expression of VEGFR-1, VEGFR-2 and NRP-1 and activation of VEGFR-2.

It is notable that, no significant change of VEGF mRNA and protein was observed before and after UVA irradiation.

### Activation of VEGFR-1 and VEGFR-2 by UVA involves PKC and SFK activation

**Figure 4:**
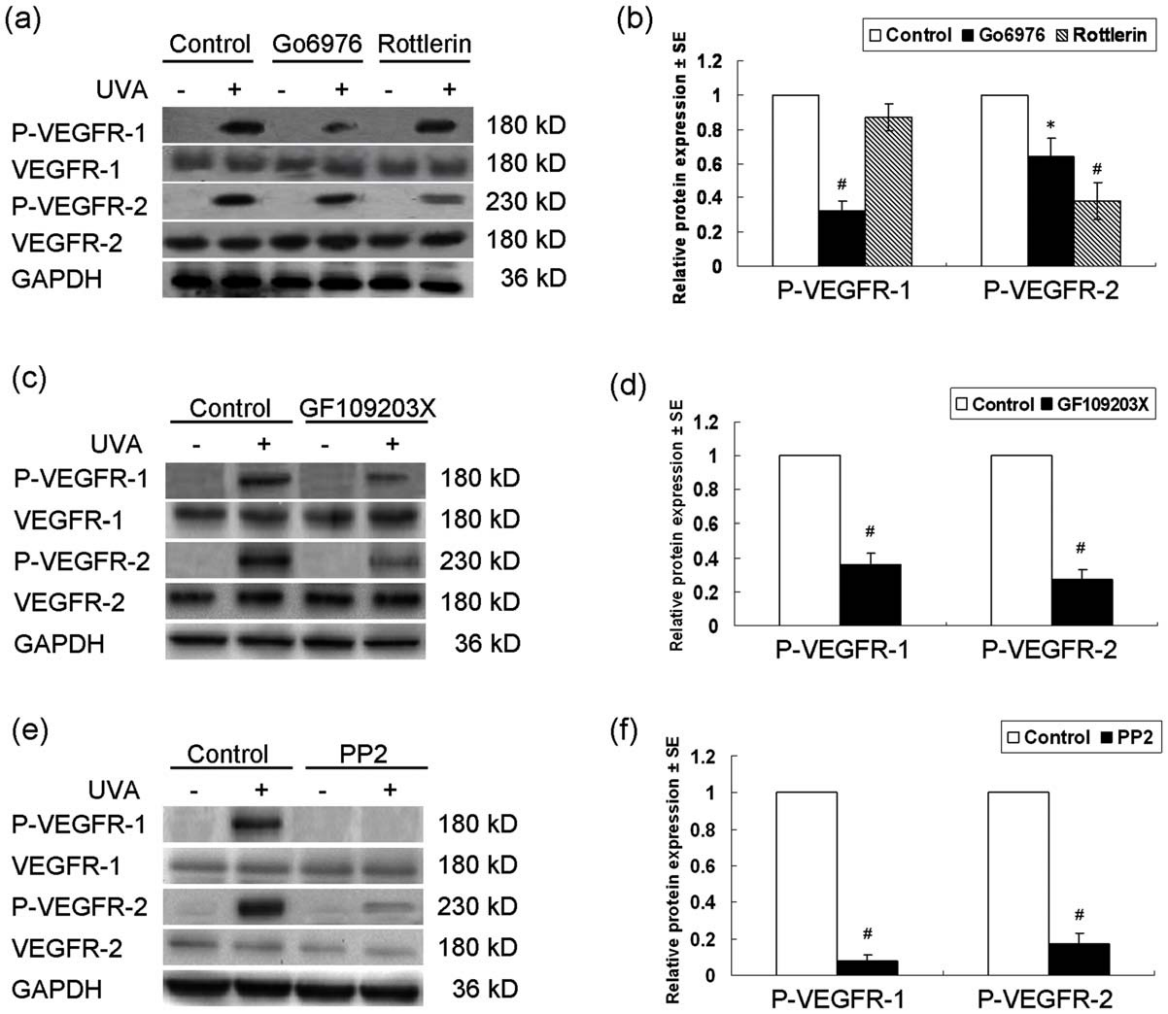
Activation of VEGFR-1 and VEGFR-2 by UVA involves activation of PKC and SFK. (a) Western blotting detection of P-VEGFR-1 and P-VEGFR-2 in keratinocytes 12 h after treatment of 10 J/cm^2^ UVA with or without pre-incubation of Go6976 (0.5 µM), or rottlerin (5.0 µM) for 1 h. (b) The densitometric analysis of P-VEGFR-1 and P-VEGFR-2 in UVA-treated groups in (a). (c) Western blotting detection of P-VEGFR-1 and P-VEGFR-2 in keratinocytes 12 h after treatment of 0 or 10 J/cm^2^ UVA with or without 1 h pre-incubation of GF109203X (3 µM). (d) The densitometric analysis of P-VEGFR-1 and P-VEGFR-2 in UVA-treated groups in (c). (e) Western blotting detection of P-VEGFR-1 and P-VEGFR-2 in keratinocytes 12 h after treatment of 0 or 10 J/cm^2^ UVA with or without 1 h pre-incubation of PP2 (10 µM). (f) The densitometric analysis of P-VEGFR-1 and P-VEGFR-2 in UVA-treated groups in (e). The relative expression was normalized to the endogenous control GAPDH. P-VEGFR-1, phospho-VEGFR-1 (Y1213); P-VEGFR-2, phospho-VEGFR-2 (Tyr1175); UVA: UVA; * *P*<0.05; # *P*<0.01.

It was reported that protein kinase C (PKC) could be induced by UVA and UVB in normal human keratinocytes [34]–[35], and that PKC had been demonstrated to be involved in the UVB-induced tyrosine phosphorylation of VEGFR-1 and VEGFR-2 [28], we thus asked whether UVA-induced tyrosine phosphorylation of VEGFRs also involved the activation of PKC. Before exposure to UVA irradiation, keratinocytes were pretreated with Go6976, a selective inhibitor for PKC-α or rottlerin, a selective inhibitor of PKC-δ, then the tyrosine phosphorylation of VEGFR-1 and VEGFR-2 was examined. Compared with the controls, pretreatment with Go6976 and rottlerin both decreased the level of P-VEGFR-2, while pretreatment with Go6976 but not rottlerin decreased P-VEGFR-1 level by UVA (Figure 4a). Pretreatment with GF109203X, a broader selective inhibitor for various isoforms of PKC, also inhibited the UVA-induced tyrosine phosphorylation of VEGFR-1 and VEGFR-2 (Figure 4c). These results suggested that UVA-induced activation of VEGFR-1 and VEGFR-2 also involved PKC.

It is demonstrated that PKC, at least the δ isoform, can be activated by Src family tyrosine kinases (SFKs) both in vitro and in vivo [36]–[37]. SFKs are also activated after UVB irradiation in keratinocytes [38]–[39]. Moreover, UVB-induced activation of VEGFRs involved SFKs [28]. We thus examined the role of SFKs in UVA-induced VEGFR-1 and VEGFR-2 phosphorylation by using PP2, a specific inhibitor for SFKs. As shown in Figure 4e, the UVA-stimulated P-VEGFR-1 and P-VEGFR-2 was almost completely abrogated in the presence of PP2. This indicated that UVA also accounted for the phosphorylation of VEGFR-1 and VEGFR-2 via SFKs and PKC.

### Activated VEGFR-1 and VEGFR-2 by UVA both contribute to the survival of keratinocytes via subsequent activation of ERK1/2 and Akt

**Figure 5:**
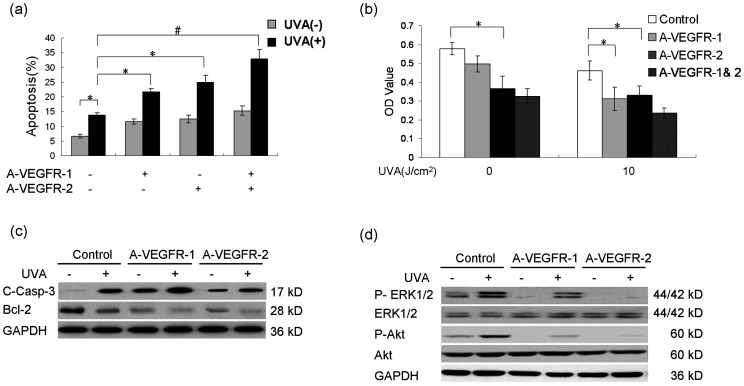
Activated VEGFR-1 and VEGFR-2 by UVA both contribute to the survival of keratinocytes via subsequent activation of ERK1/2 and Akt. (a) Keratinocytes were incubated with or without neutralizing antibodies against VEGFR-1 (A-VEGFR-1, 5 µg/ml) and VEGFR-2 (AVEGFR-2, 5 µg/ml) 24 h after treatment of 0 or 10 J/cm^2^ UVA, and cell apoptosis rate was examined by flow cytometry. (b) Keratinocytes were incubated with neutralizing antibodies against VEGFR-1 (A-VEGFR-1, 5 µg/ml) and/or VEGFR-2 (A-VEGFR-2, 5 µg/ml) 24 h after treatment of 0 or 10 J/cm^2^ UVA, and cell survival was determined by MTT assay. (c) Western blotting detection of cleaved-caspase-3 and Bcl-2 in keratinocytes treated by 10 J/cm2 UVA with or without incubation of VEGFR-1 neutralizing antibody (A-VEGFR-1, 5 µg/ml) or VEGFR-2 neutralizing antibody (A-VEGFR-2, 5 µg/ml) for 24 h. (d) Western blotting detection of phospho-ERK1/2 and phospho-Akt in keratinocytes 12 h after treatment of 10 J/cm^2^ UVA with or without pre-incubation of VEGFR-1 neutralizing antibody (A-VEGFR-1, 5 µg/ml) or VEGFR-2 neutralizing antibody (A-VEGFR-2, 5 µg/ml) for 1 h. GAPDH was served as loading control for protein normalization, UVA: UVA; * *P*<0.05; # *P*<0.01.

Moderate UVB-induced activation of VEGFR-2 played a protective role for keratinocytes [28]. Similar to UVB, UVA also increased apoptosis of keratinocytes dose-dependently (data not shown). We then examined the roles of activated VEGFR-1 and VEGFR-2 signaling in UVA-induced cell death. Compared with that treated by VEGFR-1 and/or VEGFR-2 neutralizing antibody or 10 J/cm^2^ UVA alone, VEGFR-1 and/or VEGFR-2 neutralization after 10 J/cm^2^ UVA further promoted apoptosis of keratinocytes(Figure 5a), suggesting that both the activated VEGFR-1 and VEGFR-2 signaling played an anti-apoptotic role for keratinoytes in response to UVA.

Cell survival assay showed that VEGFR-2 neutralization itself significantly reduced the survival of keratinocytes, however, neutralization of VEGFR-1 and/or VEGFR-2 could further decrease cell survival after UVA irradiation (Figure 5b), suggesting that VEGFR-2 signaling was involved in maintaining basic viability, while both VEGFR-1 and VEGFR-2 were activated and promoted cell survival in case of UVA irradiation. In addition, both VEGFR-1 and VEGFR-2 neutralization caused a further increase of cleaved caspase-3 and a decrease of Bcl-2 expression in keratinocytes on UVA exposure (Figure 5c). These data indicated that the activation of VEGFRs contributed to the survival of keratinocytes in response to UVA.

UV activates multiple signaling pathways, in which MAPK and PI3-kinase/Akt promote cell survival and function as major protectors for keratinocytes[13], [40]–[42]. Signaling via VEGFRs also induces activation of the ERK1/2 and PI3-kinase/Akt pathway [26], [43]–[44]. We thus asked whether the activation of VEGFRs by UVA could trigger ERK1/2 and Akt. As shown in Figure 5d, both ERK1/2 and Akt were activated by 10 J/cm^2^ UVA, but this effect was significantly blocked by neutralization of VEGFR-1 or VEGFR-2, suggesting that UVA-induced activation of VEGF receptors can promote survival of keratinocytes via subsequent activation of MAPK and Akt.

### Over-expressed VEGFRs in psoriatic epidermis were normalized by NB-UVB phototherapy in a way different from that by halomethasone

Psoriasis is a common, chronic, inflammatory skin disease with still unknown etiology. In our previous study, VEGFRs were found to be over-expressed in psoriatic epidermis, implicating their pathological significance in the disease [31]. As NB-UVB phototherapy is widely accepted to treat moderate-to-severe psoriasis vulgaris [45], we investigated whether these over-expressed VEGFRs in psoriatic epidermis could be affected by this therapy. For comparison, topical halomethasone monohydrate 0.05% cream, a kind of corticosteroids, was utilized as another therapy. With these therapies going on, all patients responded well without any uncomfortable complaints. Using Psoriasis Area Severity Index (PASI) scoring, we also assessed each patient's skin condition before, during and after the two therapies, with scores of 25.2±5.4 (before), l2.5±3.1 (during), and 3.6±2.1 (after) for phototherapy, and 17.0±4.0, 8.3±1.8, and 4.3±0.8 for halomethasone therapy. This result indicated that both of the two therapies were indeed effective for clinical improvement of psoriasis vulgaris (*P*<0.01).

**Figure 6:**
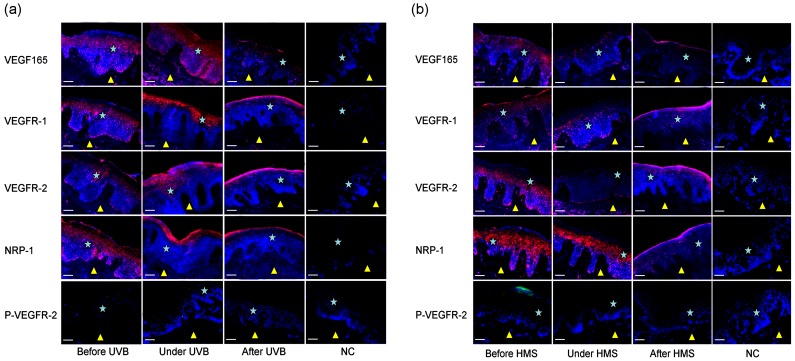
Immunofluorescence detection of VEGF165, VEGFRs and P-VEGFR-2 affected by NB-UVB phototherapy (a, skin samples from 13 patients) or halomethasone therapy (b, skin samples from 3 patients) in human psoriatic epidermis. The times of phototherapy in relation to during and after therapy were 11.2±2.2 and 26.3±3.9, and the days of halomethasone treatment in relation to during and after therapy were 12.7±2.9 and 36.0±8.0. Biopsies were taken before, during, and after phototherapy respectively. The presence of VEGF165 and VEGFRs was indicated red. The presence of P-VEGFR-2 was indicated green. The cellular nuclei were counterstained with DAPI (blue nuclear signal). P-VEGFR-2, phospho-VEGFR-2 (Tyr1175); UVB, narrowband UVB therapy; HMS, topical halomethasone monohydrate 0.05% cream; NC, negative controls, which were incubated with non-immune mouse IgG. Bars: 100 µm; Asterisk, epidermis; Yellow triangle, dermis.

In addition, the skin lesions were pathologically resolved by both of the two treatments, characterized by attenuated hyperkeratosis, parakeratosis and acanthosis of the epidermis (Figure 6). The expression of VEGF165 got even higher during phototherapy, including in the dermis, but greatly declined after therapy. Differently, the over-expressed VEGFRs in the whole psoriatic epidermis was significantly down-regulated both during and after phototherapy, and interestingly, their expression decreased in a down to top advancement way, that is, VEGFRs over-expressed in the basal layers were much more sensitive to UVB than that in upper layers, and were first downregulated in response to NB-UVB, with the UVB dose continuing to grow during phototherapy, VEGFRs over-expressed in the upper epidermis were also gradually downregulated, even to the stratum corneum after treatment. In addition, VEGFRs were activated in psoriatic epidermis, and their activation was enhanced during phototherapy in epidermis still with VEGFRs expression, but turned undetectable after the whole therapy (Figure 6a).

The overall result from treatment by halomethasone was similar to that by NB-UVB therapy. However, the process was quite different, in which VEGF165, VEGFRs and P-VEGFR-2 decreased in a gradual, homogeneous manner (Figure 6b), suggesting VEGF and its receptors are not target molecules of halomethasone, and their expression automatically declined due to improvement of the skin lesions by treatment of the drug.

## Discussion

Skin is strategically located at the interface with the external environment where it detects, integrates, and responds to a variety of stressors including UV radiation. It has been established that the skin coordinates and/or regulates not only peripheral but also global homeostasis [46]. In case of solar radiation, for example, the skin will produce and respond to CRH-POMC system, neuropeptides, neurotrophins, neurotransmitters, and other immunoregulatory molecules, underlying a role for these agents in the skin response to stress. These endocrine mediators with their receptors are organized into epidermal and dermal units that allow precise control of their activity in a field-restricted manner. The skin neuroendocrine system communicates with itself and with the systemic level through humoral and neural pathways to induce vascular, immune, or pigmentary changes, to preserve and maintain the skin structural and functional integrity and systemic homeostasis [47].

Human epidermal keratinocytes were found to functionally express VEGFRs and that VEGF/VEGFRs autocrine signaling might be responsible for the behavior of keratinocytes, as in psoriasis and cutaneous wound repair [26], [48], [49]. VEGFRs could be enhanced and activated by UVB, and that the activated VEGFR-2, but not VEGFR-1 was involved in the pro-survival mechanism [28]. However, UVA contributes 95% of UV in the sunlight, and prolonged exposure to UVA can also cause premature skin aging and skin cancer, although its underlying mechanism is still poorly understood [50].

Our present study showed that UVA could not elicite the expression of VEGF from normal human keratinocytes *in vitro* and *in vivo*. As VEGFRs were activated by UVB independent of VEGF in keratinocyte [28], we hypothesized that UVA could also exert an effect to keratinocytes by modulating VEGFRs signaling pathway. Indeed, UVA also increased the expression of VEGFRs in keratinocytes both at mRNA and protein levels, and the tyrosine phosphorylation of VEGFR-1 and VEGFR-2 was promoted by both moderate and high doses of UVA.

The tyrosine phosphorylation of VEGFRs in response to UVB could be mediated by PKC, we then asked if PKC was also involved in UVA-induced activation of VEGFRs. As a result, the activation of VEGFR-2 was mediated both through PKC-α and PKC-δ, while VEGFR-1 was activated most likely through PKC-α. A broader selective inhibitor for PKC significantly inhibited the UVA-induced activation of VEGFRs. SFKs were also found to be required in the UVA-induced activation of VEGFRs by modulating PKC. A previous study showed that UVA-induced delayed and sustained ERK activation is EGFR kinase-independent, but PLC/calcium/PKC-mediated [51]. Our results suggested that this kind of ERK activation was dependent of VEGFRs, especially VEGFR-2, providing a survival signal to normal human keratinocytes, which may serve as an important mechanism for cell transformation and potential skin carcinogenesis induced by UVA.

By immunofluorescence, we found that the expression and activation of VEGFRs was enhanced by UVA in the whole epidermis, including in the well-differentiated keratinocytes of the upper layers, implicating that the basic expression of VEGFRs is involved in cell growth, proliferation, adhesion and migration [26], [27], [52], but their over-expression and activation in the upper layers (also called spinous layer and grained layer) is to protect keratinocytes from UV-induced photodamage, as the major function of keratinocytes in those two layers is to maintain the integrity of the epidermis. In addition, we demonstrated that activation of both VEGFR-1 and VEGFR-2 signaling in response to UVA promoted survival of keratinoytes via subsequent activation of ERK and Akt. Thus, we conclude that UV-induced activation of VEGFRs is involved in pro-survival mechanism, but this mechanism may offer opportunities for malignant transformation of keratinocytes and development of non-melanoma skin cancers under long-term, chronic UV exposure.

In patients with psoriasis, VEGF is overexpressed both in serum and skin lesions, it acts as an inflammatory mediator, plays important roles in skin angiogenesis and epidermal hyperplasia [53]–[55]. VEGF is a pro-angiogenic factor and several anti-VEGF therapies are used in the treatment of diseases that are characterized by abnormal formation of blood vessels such as certain cancers and age-related macular degeneration [53]. Dysregulated angiogenesis has been observed in inflammatory diseases and might underly chronic cutaneous inflammation in psoriasis [53]. Several experimental studies and clinical reports suggest that VEGF is involved in psoriasis pathogenesis, among those, transgenic over-expression of VEGF in keratinocytes in mice resulted in skin inflammation and a phenotype resembling human psoriasis. In different psoriasis models, anti-VEGF antibody treatment of mice, already displaying disease symptoms, resulted in an overall improvement of the cutaneous lesions [53]. Finally, a patient with psoriasis was reported to had complete remission of psoriasis during bevacizumab (a monoclonal antibody against VEGF) therapy for colon cancer [56]. Therefore, VEGF is a pro-inflammatory factor in the pathogenesis of psoriasis. In addition, we previously showed that VEGFRs were also overexpressed in psoriatic epidermis [31], but their real function in psoriasis remained unclear. Based on the findings that proliferation of keratinocytes could be promoted via VEGF/VEGFRs pathway [26], [27], and that treatment with a VEGFR tyrosine kinase inhibitor could inhibit chronic and acute skin inflammation [57], we hypothesized that the overexpressed VEGFRs in psoriatic epidermis might be involved in the pathological process of psoriasis.

To date, many kinds of therapeutic ways have been adopted to treat psoriasis, among which NB-UVB phototherapy has proved to be effective and safe, and, although not completed, some mechanisms of phototherapy are being gradually discovered [58]. Thus, by using NB-UVB phototherapy or topical halomethasone monohydrate 0.05% cream, we further studied the function of VEGFRs in psoriatic epidermis. Surprisingly, the over-expressed VEGFRs in psoriatic epidermis were significantly attenuated by both of the two treatments, and that the expression of VEGFRs was positively correlated with the skin condition of patients with psoriasis. During NB-UVB therapy, VEGFRs expression declined first in the basal epidermis, and this alteration gradually occurred in the upper psoriatic epidermis. VEGFRs were activated in psoriatic epidermis, their activation was enhanced during NB-UVB treatment, but turned undetectable after whole therapy. This process was quite different from that by halomethasone, in which VEGFRs and phospho-VEGFRs decreased in a gradual, homogeneous manner. This phenomenon, combined with our previous findings, highly suggest that over-expressed VEGFRs played an important role in epidermal hyperplasia, and they might be degraded by gradually increasing UVB doses during phototherapy. VEGFRs over-expressed in the basal cell layer were much more sensitive to UVB and were degraded earlier than that in upper layers, a time when VEGFRs were still over-expressed in upper layers to maintain the integrity of the epidermis. With the UVB doses continuing to increase, VEGFRs were degraded in the whole psoriatic epidermis, resulting in a normalization of the epidermal hyperplasia. The whole process caused the phenomenon that the location of VEGFRs shafted to the upper epidermis, and to the stratum corneum after NB-UVB treatment.

We previously showed that moderate dose UVB-induced upregulation of VEGFRs was mediated through hypoxia and oxidative stress [28]. Interestingly enough, hypoxia and oxidative stress also existed in the psoriatic lesions [59], [60], which might be associated with overexpression of VEGFRs and subsequent hyperplasia of the epidermis. So, by treatment of halomethasone, the conditions of lesional inflammation, hypoxia and oxidative stress were gradually relieved, which eventually brought about remission of the disease. In the meantime, the expression of VEGFRs declined spontaneously. So, we infer that although VEGFRs are not an initial factor, they really act as a key intermediate in psoriasis pathogenesis, and treatments targeting VEGFRs would be of potential significance for psorisis.

In summary, our data demonstrated that UVA enhanced the expression and activation of VEGFRs in keratinocytes, and the activated VEGFR-1 and VEGFR-2 both contributed to the survival of keratinocyes, suggesting an important role of VEGFRs as a pro-survival factor for keratinocytes on UV exposure. In additon, VEGFRs may be involved in the pathological process of psoriasis, and NB-UVB phototherapy is effective for psoriasis by directly down-regulating the overexpressed VEGFRs in psoriatic epidermis.
